# Hybridization between two bitterling fish species in their sympatric range and a river where one species is native and the other is introduced

**DOI:** 10.1371/journal.pone.0203423

**Published:** 2018-09-07

**Authors:** Yohsuke Uemura, Shotaro Yoshimi, Hiroki Hata

**Affiliations:** 1 Department of Biology, Faculty of Science, Ehime University, Matsuyama, Ehime Japan; 2 Graduate School of Science and Engineering, Ehime University, Matsuyama, Ehime, Japan; SOUTHWEST UNIVERSITY, CHINA

## Abstract

The distributions of two bitterling fish (subfamily: Acheilognathinae), *Tanakia lanceolata* and *T*. *limbata*, overlap in western Japan. Acheilognathinae fish lay their eggs in the gills of freshwater bivalves, and the early juvenile stage develops in the gills. Populations of freshwater bivalves are declining worldwide, which has limited the number of spawning substrate for bitterlings. *T*. *limbata* has been artificially introduced to some rivers in Ehime, Japan, where it coexists with native *T*. *lanceolata*, and some hybrids have been observed. We collected both species from several sites in western Japan, and from the Kunichi River system in Ehime, and analyzed genetic population structure based on six microsatellite loci and sequences of the mitochondrial cytochrome *b* gene. Structure analysis identified three genetically distinct populations: *T*. *lanceolata*, *T*. *limbata* “West Kyushu”, and *T*. *limbata* “Setouchi”. Two clades of *T*. *limbata* were also supported by molecular phylogenetic analyses based on cytochrome *b*. Hybrids in Ehime originated mostly from interbreeding between male *T*. *lanceolata* and female *T*. *limbata* “West Kyushu”, and made up 10.2% of all collected fish, suggesting that hybrids occurred frequently between females of colonizing species and males of native species. On the other hand, interspecific hybrids were detected at rates of 40.0%, 20.0%, and 17.6% in the Ima River (Fukuoka), Midori River (Kumamoto), and Kase River (Saga), respectively, which are naturally sympatric regions. We found a few *T*. *limbata* “Setouchi” in the Midori and Kase Rivers, which were supposed to be introduced from other regions, coexisting with native *T*. *limbata* “West Kyushu”, and this cryptic invasion may have triggered the interspecific hybridization. These results suggest that artificial introduction of a fish species, a decline in the unionid population, and degradation of habitat have caused broad hybridization of bitterlings in western Japan.

## Introduction

Artificially transplanting species can trigger the extinction of native species not only through cascade effects in prey–predator interactions but also through unexpected hybridization; that is, invasive hybridization [1,2]. *Tanakia lanceolata* is distributed throughout Japan except on Hokkaido Island and southern Kyushu Island, whereas *T*. *limbata* is found in western Honshu, northern Shikoku, and northern Kyushu [3]. In Japan, 15 of the 16 native bitterling species are listed on the Japanese Red Lists and are facing extinction crises [4]. Bitterlings deposit their eggs in the gills of freshwater bivalves [5,6], and obligately depend on the bivalves for reproduction.

Only *T*. *lanceolata* occurred exclusively in the Matsuyama Plain, Ehime, but *T*. *limbata* was first discovered in this area in 1979 [7]. Molecular phylogenetic analyses of the bitterlings have revealed that *T*. *limbata* in Ehime originated from western Kyushu [8,9], and hybridization is suspected between the two species. These two species diverged about 10 million years ago [10], which is the same time that cutthroat trout (*Oncorhynchus clarkii*) and rainbow trout (*O*. *mykiss*), the most famous invasive hybridization system in western North America, diverged [11]. The two bitterling species have fertile hybrids [12], but their survival rate in the post-larval stage is lower than that of purebred fish, suggesting the presence of postzygotic isolation [13]. Prezygotic isolation has also likely been established through sexual isolation between hybridizing fishes because of a decline in parental fitness of that breed with other species in their sympatric range [14,15]. In fact, habitat segregation and different preference for breeding substrate within a river has been observed between *T*. *lanceolata* and *T*. *limbata*. That is, *T*. *lanceolata* lives extensively in rivers and spawns mainly on unionids in the thalweg section with strong flow, whereas *T*. *limbata* lives on the bank side of rivers with weak flow, and spawns on unionids near the banks [16]. Additionally, male *T*. *limbata* defends a unionid as a breeding territory against *T*. *lanceolata* as well as males of *T*. *limbata* [17,18].

The unionid mussels *Pronodularia japanensis* and *Nodularia douglasiae nipponensis*, which serve as the spawning substrate for bitterlings on the Matsuyama Plain, have decreased rapidly over the past 30 years because of habitat degradation [19]. In Japan, rivers and agricultural channels, which are the main bitterling habitats, have been degraded by urbanization and fragmented by weirs; many banks and river beds have been covered with concrete, as seen in Matsuyama Plain. This has reduced the population density of freshwater unionids on Kyushu Island, leading to decreases in the population of bitterlings [20]. In addition, these conditions may force group spawning by multiple males and females instead of pair spawning as seen in a bitterling, *Rhodeus ocellatus* [21]. In this group spawning, different species of bitterling fish may spawn simultaneously and hybridize incidentally with each other [22].

In fact, the endemic Japanese bitterling, *Rhodeus ocellatus kurumeus*, inhabits small ponds and agricultural channels throughout western Japan [23,24], and *R*. *o*. *ocellatus* was introduced into Japan in the 1942 [25,26]. Since then, most of the *R*. *o*. *kurumeus* populations have become extinct because of competition and introgressive hybridization between these two species [27].

Therefore, it is important to determine the occurrence and frequency of hybrids and genetic introgression between *T*. *lanceolata* and *T*. *limbata* in various rivers in western Japan where the two species originally coexisted and in the river in Matsuyama where *T*. *limbata* was introduced. If hybridization has occurred in some rivers in western Japan, it will be necessary to survey their genotypes to detect their origin and consider whether artificial transplantation is the cause of the hybridization, as suggested for the Matsuyama Plain.

For this purpose, we classified individuals as *T*. *lanceolata*, *T*. *limbata*, or their hybrid at the individual level using microsatellite nuclear DNA markers. We also conducted phylogeographic analyses based on cytochrome *b* (cyt *b*) sequences in mitochondrial DNA to clarify the population structure of these two species and their hybrids in western Japan.

## Materials and methods

### Ethics statement

The field sampling and sample treatment were conducted in accordance with the “Guidelines for the use of fishes in research” by the Ichthyological Society of Japan (http://www.fish-isj.jp/english/guidelines.html). All animal experiments were approved by the Ethics Committee for Animal Experiments of Ehime University. The experimental procedures were conducted in accordance with the approved guidelines.

### Sample collection

**Fig 1:**
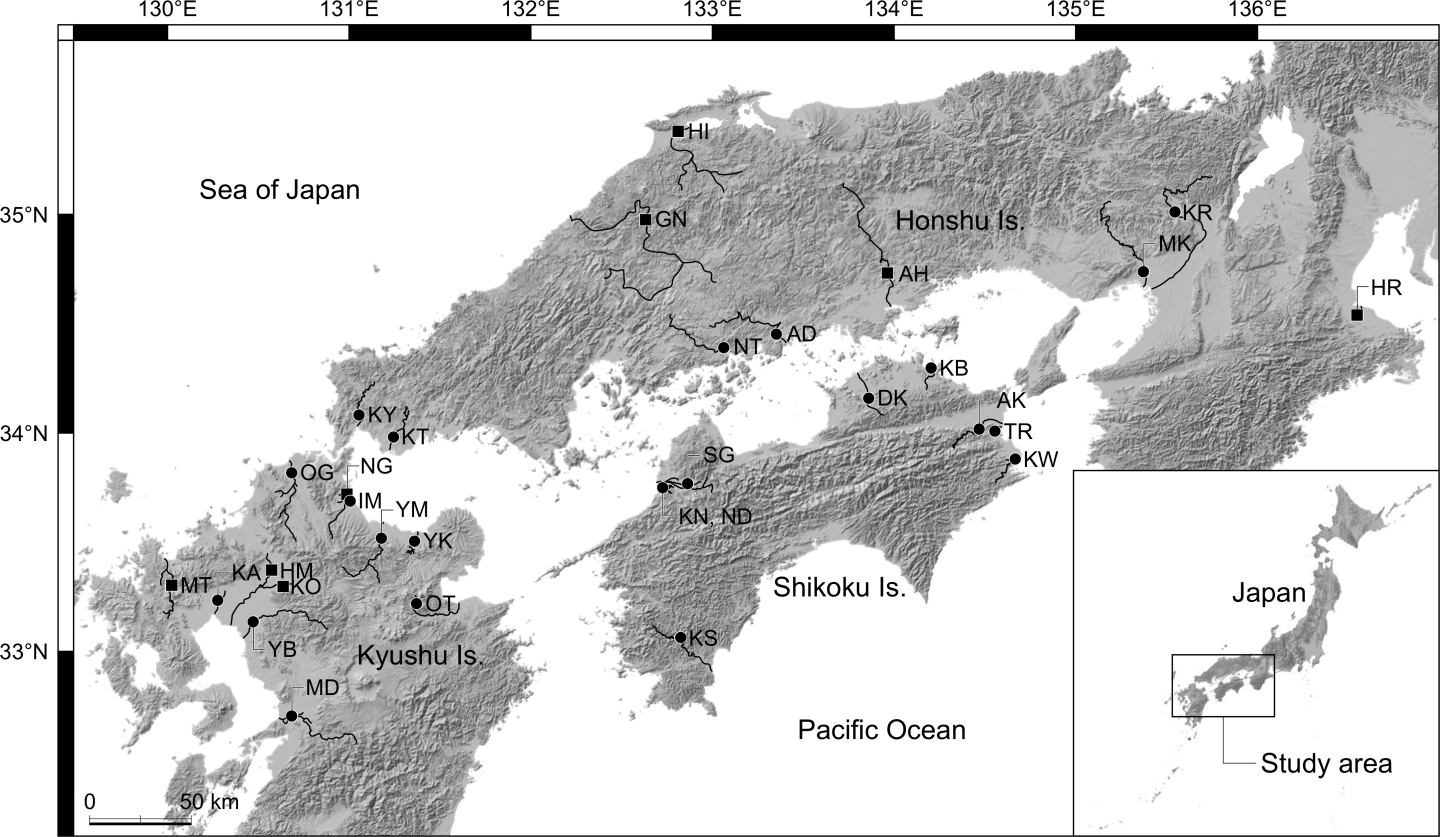
Map showing the study sites in western Japan. Abbreviations for the river names correspond to those given in S1 Table. Circles and squares represent localities where fish specimens were collected for this study and localities where sequences are cited from the DDBJ, respectively. These shaded-relief maps are made by ourselves base on maps provided under CC BY 4.0 by the Geospatial Information Authority of Japan (https://maps.gsi.go.jp).

**Fig 2:**
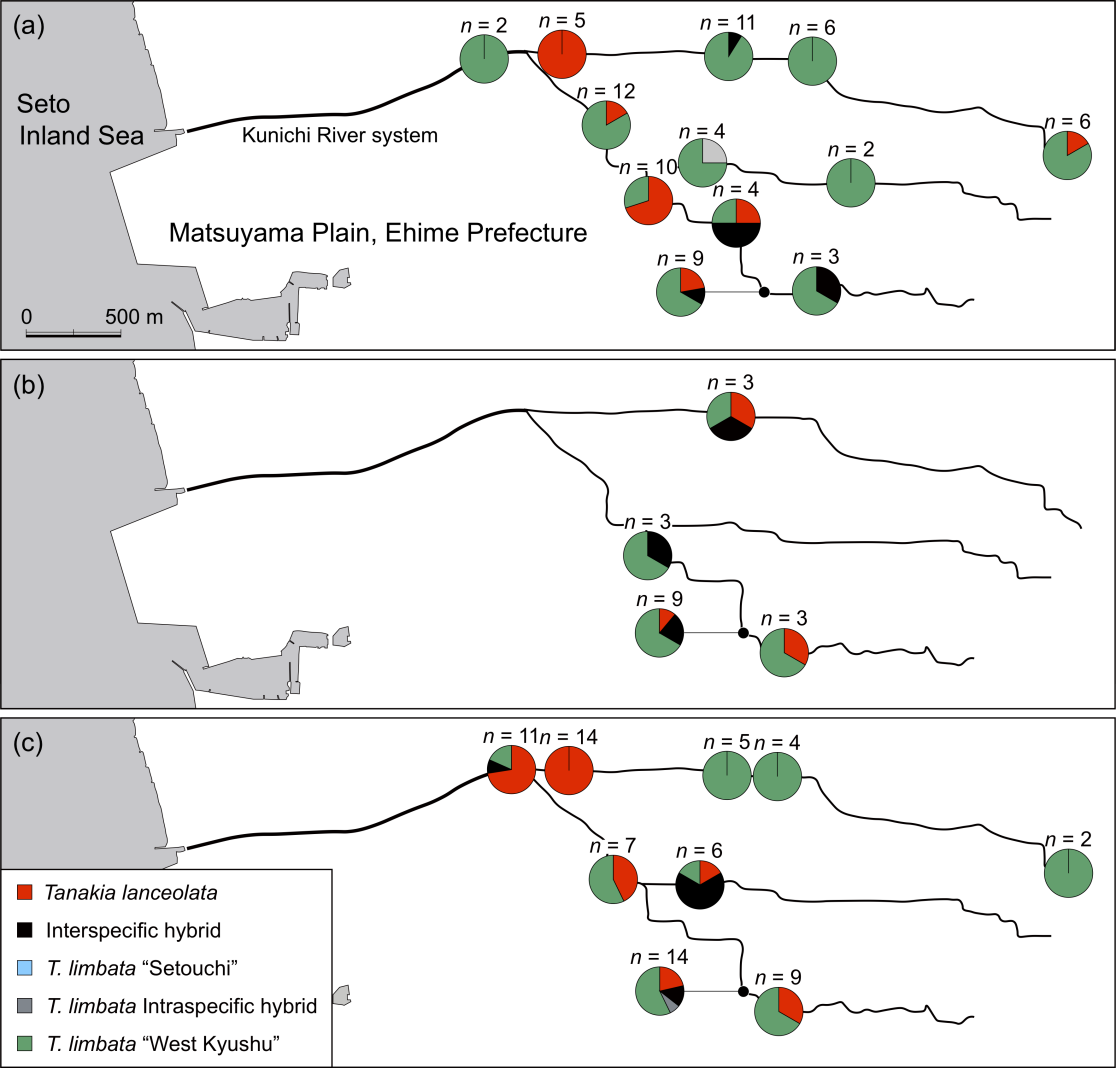
**Genotype composition of *Tanakia* species in the Kunichi River system, Matsuyama, Ehime, (a) in June, (b) August, and (c) October 2014.** The maps are made by ourselves base on a map provided under CC BY 4.0 by the Geospatial Information Authority of Japan (https://maps.gsi.go.jp).

We collected *T*. *lanceolata*, *T*. *limbata*, and their putative hybrids in western Japan between October 2011 and October 2014 using minnow traps (Fig 1; S1 Table). In particular, we collected specimens from nine rivers (Midori River, Kumamoto Prefecture; Kase River, Saga Pref.; Onga River and Ima River, Fukuoka Pref.; Yakkan River, Oita Pref.; Koto River, Yamaguchi Pref.; Ashida River, Hiroshima Pref.; Muko River, Hyogo Pref.; and Katsura River, Kyoto Pref.) where these two species originally coexisted. No permission for trapping of these fishes was required from the authorities because these bitterling populations were not endangered or commercially important. The collected specimens were sacrificed by immersion in ice-water slurry, and then fixed in 99% ethanol and brought to the laboratory. Some specimens were used for molecular phylogenetic analyses based on cyt *b* in Matsuba et al. [9]. We collected *T*. *lanceolata* and *T*. *limbata* and the putative hybrids between the two species using an electrofishing unit (LR-24, Smith-Root Inc., Vancouver, WA, USA) in the Kunichi River system, Matsuyama Plain, Ehime Pref. in June, August, and October 2014 (Fig 2). The permission was granted by Ehime Prefecture (H26-4) for this sampling. We cut part of their dorsal or caudal fin, and released them. These fin clips were preserved in 99% ethanol.

### Genotyping

We determined the genotype of the specimens using microsatellite markers. Samples of the dorsal fin or caudal fin (ca. 2 × 2 mm^2^) were excised from the ethanol-preserved specimens. Total genomic DNA was extracted using the genomic DNA purification kit (Promega, Madison, WI, USA) following the manufacturer’s protocol. Microsatellite analyses was performed at two loci (*RC363* and *RC317A*), which were reported for *Rhodeus ocellatus* by Shirai et al. [28] and four loci (*Rser02*, *Rser03*, *Rser07*, and *Rser10*) reported for *Rhodeus sericeus* by Dawson et al. [29]. We amplified the target sequences via polymerase chain reaction (PCR) using tagged primers and tagged fluorescent dye labels as follows, following the methods of Schuelke [30]: *RC363*, *Rser07*, and PET; *RC317A*, *Rser03*, and FAM; *Rser02* and NED; and *Rser10* and VIC. PCR reactions were conducted using 3.15 μL distilled water, 0.15 μL 50 mM MgCl_2_, 0.5 μL 10× ImmoBuffer, 0.5 μL 10 mM dNTP mix, 0.02 μL 10 μM forward primer, 0.08 μL 10 μM reverse primer, 0.08 μL 10 μM fluorescent dye label, 0.025 μL BIO TAQ HS DNA Polymerase (Bioline, London, UK), and 0.5 μL DNA template. We analyzed the length of the fragments using the ABI 310 Genetic Analyzer (Thermo Fisher Scientific, Waltham, MA, USA) and Peak Scanner version 2.0 (Thermo Fisher Scientific). Micro-Checker version 2.2.3 was used to check for null alleles [31]. No null alleles were found in any marker, and we used all markers for further analyses. Private alleles for each maker were detected by GenAlEx 6.5 [32].

The population genetic structure of these bitterling species was analyzed using Structure version 2.3.4 [33]. The Structure parameters were set as follows: 50,000 burn-in, 200,000 Markov chain Monte Carlo steps, admixture model with independent allele frequencies, and 10 replicates of each simulation from *K* = 1 to 10 genetic clusters. The optimum *K* value was three using Structure Harvester version 0.6.94 ([34], S1 Fig). We detected attributions of these individuals to three populations: *T*. *lanceolata*, *T*. *limbat*a “West Kyushu”, and *T*. *limbat*a “Setouchi”. We defined the hybrids between populations as having more than 20% of two types of genetic components [35]. Each individual was also assigned to one of six genotypic classes based on the posterior probabilities: two parental species (P0, P1), first-generation hybrid (F1), second-generation hybrid (F2), backcross of F1 with P0, and backcross of F1 with P1, using by NewHybrids [36]. Parameters of NewHybrids were set as follows: without individual or allele frequency prior information, Jeffreys like prior for both mixing proportions and allele frequencies, 25,000 sweeps of burn-in, and 100,000 iterations of the Markov chain Monte Carlo. Expected heterozygosity (*He*) and observed heterozygosity (*Ho*) of each study site were determined using Arlequin 3.5.1.3 [37].

### Molecular phylogeny based on the mitochondrial cyt *b* gene

The cyt *b* gene region of mitochondrial DNA (mtDNA) was amplified using the forward primer NEW-FOR, 5’-AGCCTACGAAAAACACACCC- 3’ [38] and the reverse primer, cytb-Rev, 5’-GATCTTCGGATTACAAGACC-3’ [8]. Each PCR was performed in a 10 μL reaction volume containing 3.35 μL sterile distilled water, 5.0 μL Ampdirect Plus (Shimadzu, Kyoto, Japan), 0.3 μL each primer (10 μM solutions), 0.05 μL *Taq* DNA polymerase (BIOTAQ HS DNA Polymerase, Bioline), and 1 μL template. The thermal cycle profile was as follows: initial denaturation at 94°C for 10 min, 30 cycles of 94°C for 30 s, 58°C for 1 min, 72°C for 1.5 min, and a final extension at 72°C for 10 min. The PCR products were purified using polyethylene glycol following a published protocol [39] and subjected to direct cycle sequencing employing BigDye Terminator version 3.1 (Thermo Fisher Scientific) using the PCR primers. The sequencing protocol was that recommended by ABI. Labeled fragments were sequenced on an ABI 3130 platform (Thermo Fisher Scientific). Forward and reverse sequences were assembled using Geneious version 7.1.7 [40]. The assembled sequences were translated into proteins and aligned.

Then the protein sequences were reverse-translated prior to the phylogenetic analyses using MEGA 6.06 software [41]. We ran χ^2^ homogeneity tests to explore the compositional homogeneity of the nucleotides of all codons of the cyt *b* gene. These tests showed no significant heterogeneity in terms of base frequency. Phylogenetic trees were constructed using both the maximum likelihood (ML) and Bayesian inference (BI) methods. We calculated Akaike information criterion values and selected the best substitution model using Kakusan 4 software [42]. We employed the GTR+G model when evaluating all three codon positions by the ML method, and the K80+G, GTR+G, and GTR+G models to evaluate the first, second, and third codon positions, respectively, when applying the BI method. ML and BI analyses were performed using RAxML version 8 [43] and MrBayes version 3.2.6 [44] software, respectively. Two independent Markov-chain Monte Carlo runs, each of 2,000,000 generations, were performed for the BI method; the trees were sampled every 100 generations. The first 2,001 trees were discarded as burn-in. A majority-rule consensus tree was constructed from the remaining 18,000 trees. We confirmed that all analyses attained the stationary condition well before the end of the burn-in period. To this end, we plotted the natural logarithms of the likelihoods of all sampled trees against the generation time, and confirmed that the average standard deviation of split frequencies and the potential scale reduction factor for all parameters reached 0.006 and 1.00, respectively. We also confirmed that the effective sample size values for all parameters were more than 200.

For genotyping of mtDNA of all specimens, we amplified cyt *b* region of all specimens as described above, and restriction fragment analysis was conducted. An endonuclease, *Hha*I (TaKaRa Bio, Shiga, Japan), was used for productions of species-specific restriction fragment length polymorphism (RFLP) with incubation at 37°C for 2 hours in buffer recommended by the manufacturer [45]. PsiI (New England Biolabs, MA, USA) was used to detect *T*. *limbat*a “West Kyushu” and *T*. *limbat*a “Setouchi”. Restriction fragments were electrophoresed on 3% Agarose (NIPPON Genetic, Tokyo, Japan) at 100V for 30 min. *Hha*I produced fragments of 91, 218, 252 and 369 bps for *T*. *lanceolata*, and fragments of 18, 109, 252 and 821 bps for *T*. *limbata*. PsiI produced fragments of 364 and 818 bps for *T*. *limbat*a “West Kyushu” and fragments of 307, 364 and 511 bps for *T*. *limbat*a “Setouchi”.

## Results and discussion

**Fig 3:**
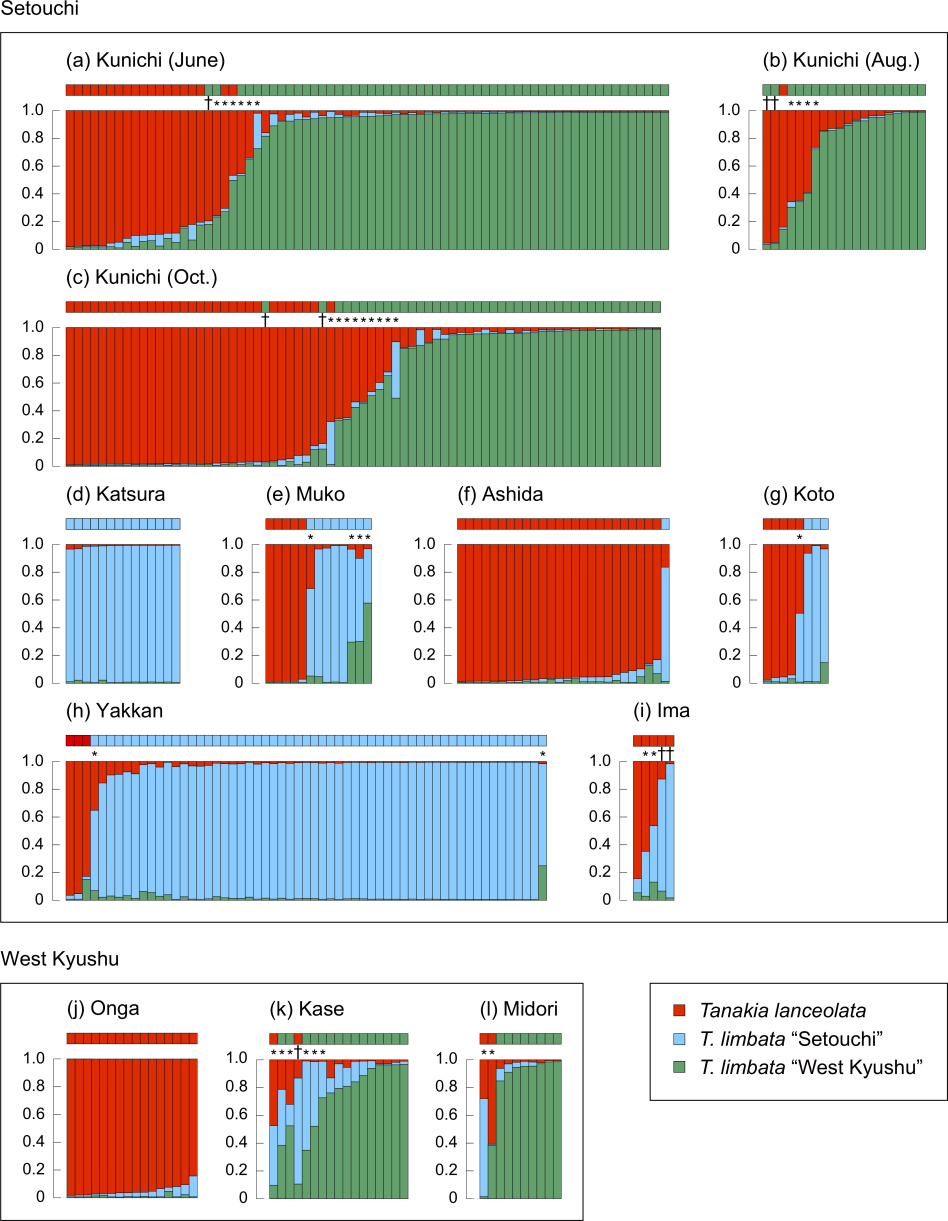
Result of structure analysis and mitochondrial genotype of *Tanakia lanceolata*, *T*. *limbata*, and their hybrids collected in western Japan based on six microsatellite loci. (a) Kunichi River system in Ehime Pref. in June, (b) Kunichi River system in August, (c) Kunichi River system in October, (d) Katsura River in Kyoto Pref., (e) Muko River in Hyogo Pref., (f) Ashida River in Hiroshima Pref., (g) Koto River in Yamaguchi Pref., (h) Yakkan River in Oita Pref., (i) Ima River in Fukuoka Pref., (j) Onga River in Fukuoka Pref., (k) Kase River in Saga Pref., (l) Midori River in Kumamoto Pref. Squares on the bars indicate mitochondrial genotype. * denotes hybrid individuals, † denotes individuals with inconsistent nuclear and mitochondrial genotypes.

**Fig 4:**
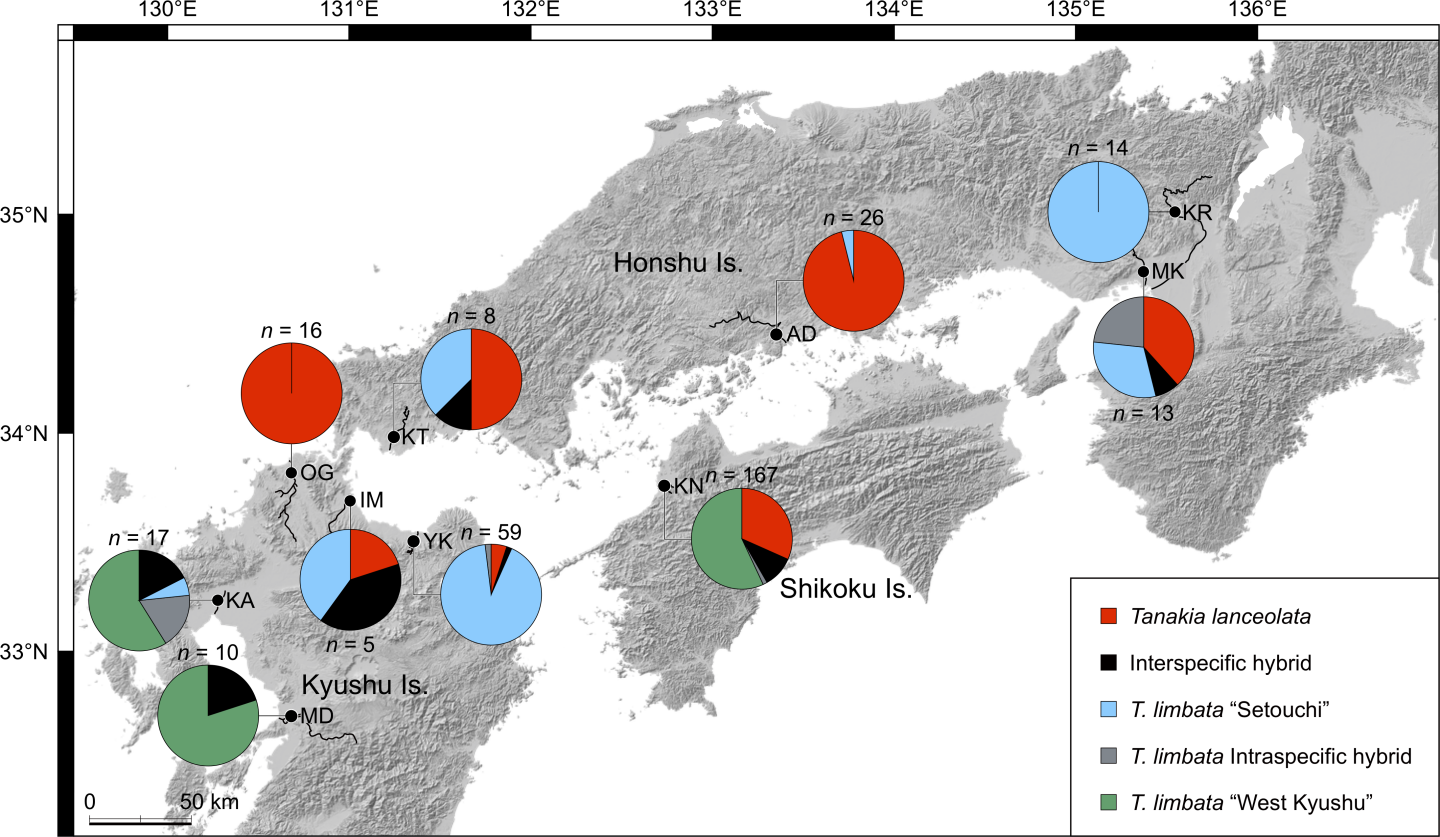
Genotype composition of *Tanakia lanceolata*, *T*. *limbata*, and their hybrids in western Japan based on six microsatellite loci. Hybrids are detected by Structure analysis based on microsatellite markers as shown in Fig 3. The shaded-relief map is made by ourselves base on a map provided under CC BY 4.0 by the Geospatial Information Authority of Japan (https://maps.gsi.go.jp).

The three populations were detected by Structure analysis using six microsatellite markers (S2 and S3 Tables; S2 Fig). Each individual was classified as *T*. *lanceolata*, *T*. *limbata* “West Kyushu”, *T*. *limbata* “Setouchi”, interspecific hybrid, or a *T*. *limbata* intraspecific hybrid (Fig 3). Based on NewHybrids, individuals were classified into three genotypic classes, *T*. *lanceolata*, *T*. *limbata*, and their F2 hybrids, and their results were mostly compatible with each other (S4 and S5 Tables). Structure Harvester detected the optimal *K* = 3, therefore we focus on the result of Structure hereafter. Interspecific hybrids were found at all sites except Ashida River and Onga River, and the proportions of interspecific hybrids were 40.0% in Ima River, 20.0% in Midori River, 17.6% in Kase River, 12.5% in Koto River, 10.2% in Kunichi River system, 7.7% in Muko River, and 1.7% in Yakkan River (Fig 4). Although the hybrid ratios may have been overestimated because of the low number of microsatellite loci, these results suggest that hybridization between *T*. *lanceolata* and *T*. *limbata* has occurred even in their sympatric range. The numbers of interspecific hybrid individuals were 1–3 out of 5–59 individuals collected from western Japan, but in Ehime, 17 hybrid individuals were found among 167 collected specimens. Hybrids in Ehime had the mitochondrial genotype of *Tanakia limbata* “West Kyushu” (14 individuals) more frequently than that of *T*. *lanceolata* (3 individuals, binomial test, *p* < 0.05), suggesting that the mtDNA introgression was bidirectional, but skewed as the introduced species play as female in this hybridization. This bias seems to be caused by the rareness of introduced species comparing with native species especially during colonizing [46], and therefore colonizing females may relax their mate choice to avoid gamete losses under the scarcity of conspecific males [47,48]. The same sex-bias in hybridization has been observed between Atlantic salmon and brown trout, and the all hybrids between these two species were offspring of colonizer females and resident males [48]. Further, five individuals were found having *T*. *lanceolata* nuclear genotype and *T*. *limbata* “West Kyushu” mitochondrial genotype (Fig 3; S4 Table). These individuals are suggested to be backcrosses between hybrids and native *T*. *lanceolata*, implying that genetic introgression is spreading via fertile F1 hybrids [49].

**Fig 5:**
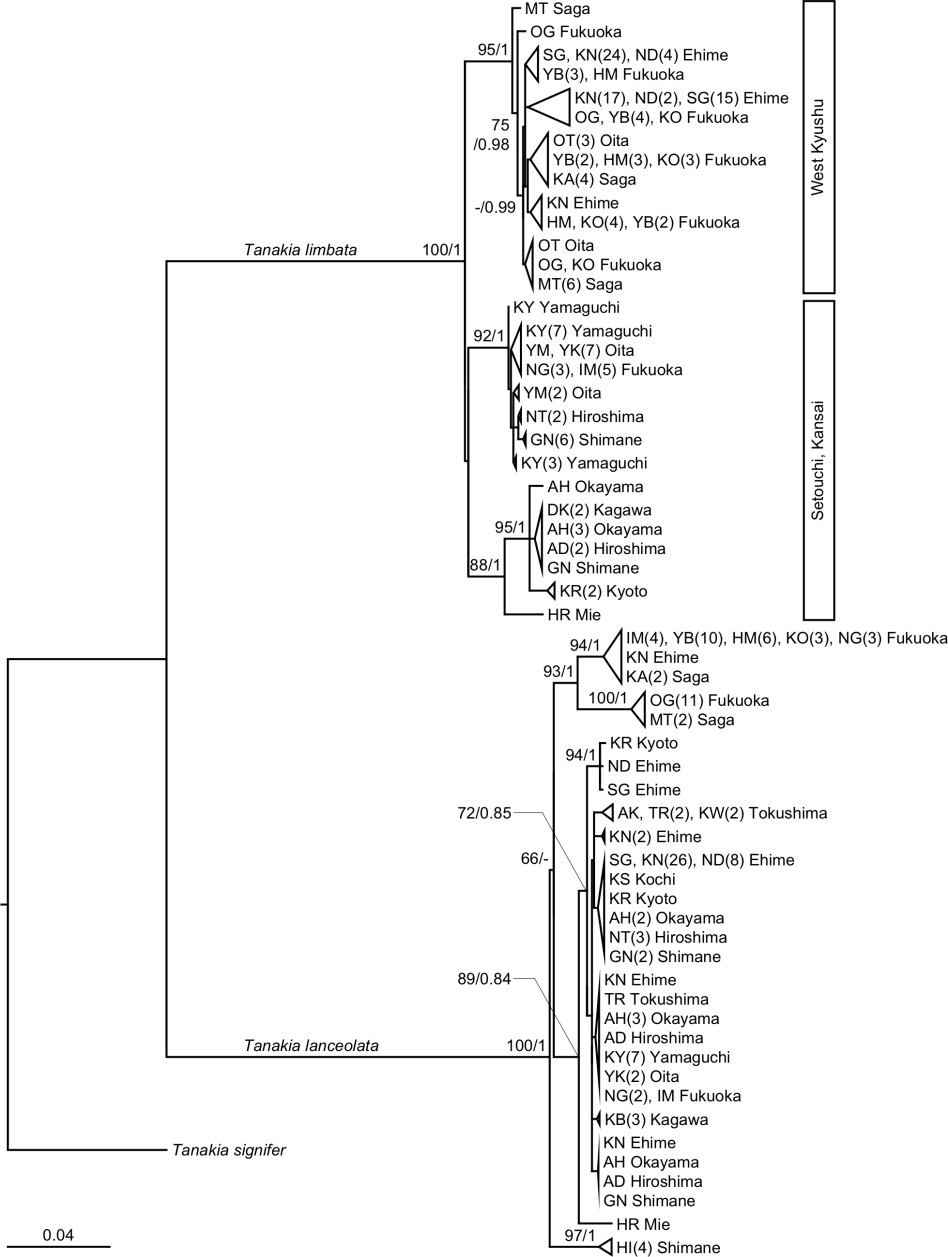
Maximum likelihood (ML) tree of *Tanakia lanceolata* and *T*. *limbata* in western Japan based on 1,027 bps of mitochondrial cyt *b*. Numbers on branches are bootstrap values (shown only above 60%) of ML and Bayesian posterior probability. Abbreviations denote rivers shown in Fig 1 and S1 Table.

*T*. *limbata* in western Japan contained two clades: the West Kyushu clade and the Setouchi clade, based on microsatellite analyses as well as a phylogeny using cyt *b* (Fig 5, [9]). This genetic differentiation between the western Kyushu and Setouchi region has also been reported in another Japanese cyprinid species, *Ospariichthys platypus* [50], which suggests a common gap that causes geographic isolation between these regions. The *T*. *limbata* “West Kyushu” individuals found in the Kunichi River system were likely introduced from west Fukuoka [9]. Furthermore, a genetic admixture of two *T*. *limbata* clades occurred in the Kase, Muko, and Yakkan rivers (Figs 3 and 4), which suggests artificial introduction over a geographic gap, that is, a case of cryptic invasions [51]. Bitterling fishes are commercially traded as aquarium fish in Japan [52], and it is a possible introduction vector. In this case, cryptic invasion causes both intraspecific hybridization with native population, and interspecific hybridization with closely related species. Artificial introduction and consequent disturbance in the population structure at these three sites possibly enhanced hybridization between *T*. *lanceolata* and *T*. *limbata*, as observed with trout on Vancouver Island, Canada [53]. By contrast, a few *T*. *lanceolata* and *T*. *limbata* hybrids were also found in the original sympatric range of the Koto and Ima rivers, where artificial introduction of these bitterlings was not observed. This observation shows that not only artificial introduction, but degradation of habitat and spawning substrate are suspected causes of interspecific hybridization in bitterlings. River branches in the Kase River system in Saga Prefecture are connected by numerous creeks stretching in a finely meshed pattern and are a suitable habitat for freshwater fishes, but many of these creeks have been covered by concrete, which has caused a decline in fishes and unionids [20,54]. The unionoid population in the Kunichi River system has been rapidly declining for the past 30 years [19]. Therefore, unionids are overused as spawning substrate for bitterlings, and simultaneous spawning may occur on the same unionids by multiple species of bitterlings, causing hybridization. Declining unionoid populations have also been reported at several other sites around Japan [55].

The distributions of *T*. *lanceolata*, *T*. *limbata*, and their hybrids in the Kunichi River system were relatively stable among seasons, and both species and their hybrids broadly inhabited this river even during their breeding season (Fig 2). *T*. *lanceolata* has been rapidly declining in the Matsuyama Plain [9]. By contrast, the density and habitat of introduced *T*. *limbata* are increasing, and has expanded into all of the native *T*. *lanceolata* habitat. In addition, hybrid individuals have appeared throughout the river, which suggests that the native *T*. *lanceolata* population is being threatened through genetic introgression and competition for resources with introduced *T*. *limbata* and hybrids.

Therefore, it is necessary to rapidly construct a conservation area where a genetically intact *T*. *lanceolata* population is maintained by removing *T*. *limbata* and the hybrids to conserve native *T*. *lanceolata* in Matsuyama. This study provides a method to distinguish among native *T*. *lanceolata*, introduced *T*. *limbata*, and their hybrids based on genetic markers, that is crucial for effective management action [51]. Further, this study has revealed that recent hybridization between *T*. *lanceolata* and *T*. *limbata* occurs widely in western Japan, which suggests the need to conserve unionid bivalves, which serve as the sole spawning substrate for bitterlings, and to restrict artificial introduction of bitterlings to prevent further cryptic invasion and consequent invasive hybridization.

## Supporting information

S1 Fig Delta *K* values calculated by Evanno method using Structure Harvester v0.6.94. (TIF)

S2 Fig Frequency of alleles of six microsatellite markers. (TIF)

S1 Table Study specimens of *Tanakia lanceolata*, *T*. *limbata*, and their hybrids collected in western Japan. The sampling localities, number of individuals, and accession numbers of the cytochrome *b* gene. Sequences with accession numbers in bold were deposited for this study, and the other sequences are cited from the DDBJ.–represents this locality is out of the species range. R.S., River system; R., River; St., Stream; C., Creek; Sp., Spring.
(XLSX)

S2 Table Allelic variability at six microsatellite loci in populations of *Tanakia lanceolata*, *T*. *limbata* “West Kyushu”, *T*. *limbata* “Setouchi”, and their intraspecific and interspecific hybrids in western Japan. *Ho*, observed heterozygosity; *He*, expected heterozygosity. Results are only shown for polymorphic loci.
(XLSX)

S3 Table Private alleles of each lineage and their frequencies. (XLSX)

S4 Table Mitochondrial genotype, and nuclear genotypes based on structure and NewHybrids analyses. lan, lim_K, lim_S indicate *Tanakia lanceolata*, *T*. *limbata* “West Kyushu”, *T*. *limbata* “Setouchi”, respectively. Strains in bold font indicate translocated strains. * denotes hybrid individuals based on structure analysis, † denotes individuals with inconsistent nuclear and mitochondrial genotypes.
(XLSX)

S5 Table Genotypes of hybrids and individuals with inconsistent nuclear and mitochondrial genotypes. lan, lim_K, lim_S indicate *Tanakia lanceolata*, *T*. *limbata* “West Kyushu”, *T*. *limbata* “Setouchi”, respectively. Strains in bold font indicate translocated strains.
(XLSX)
