# Supplementary figures and images for: Hybridization between two bitterling fish species in their sympatric range and a river where one species is native and the other is introduced
Source: PLoS One. 2018 Sep 7;13(9):e0203423. doi: 10.1371/journal.pone.0203423 (PMC6128550; doi:10.1371/journal.pone.0203423)

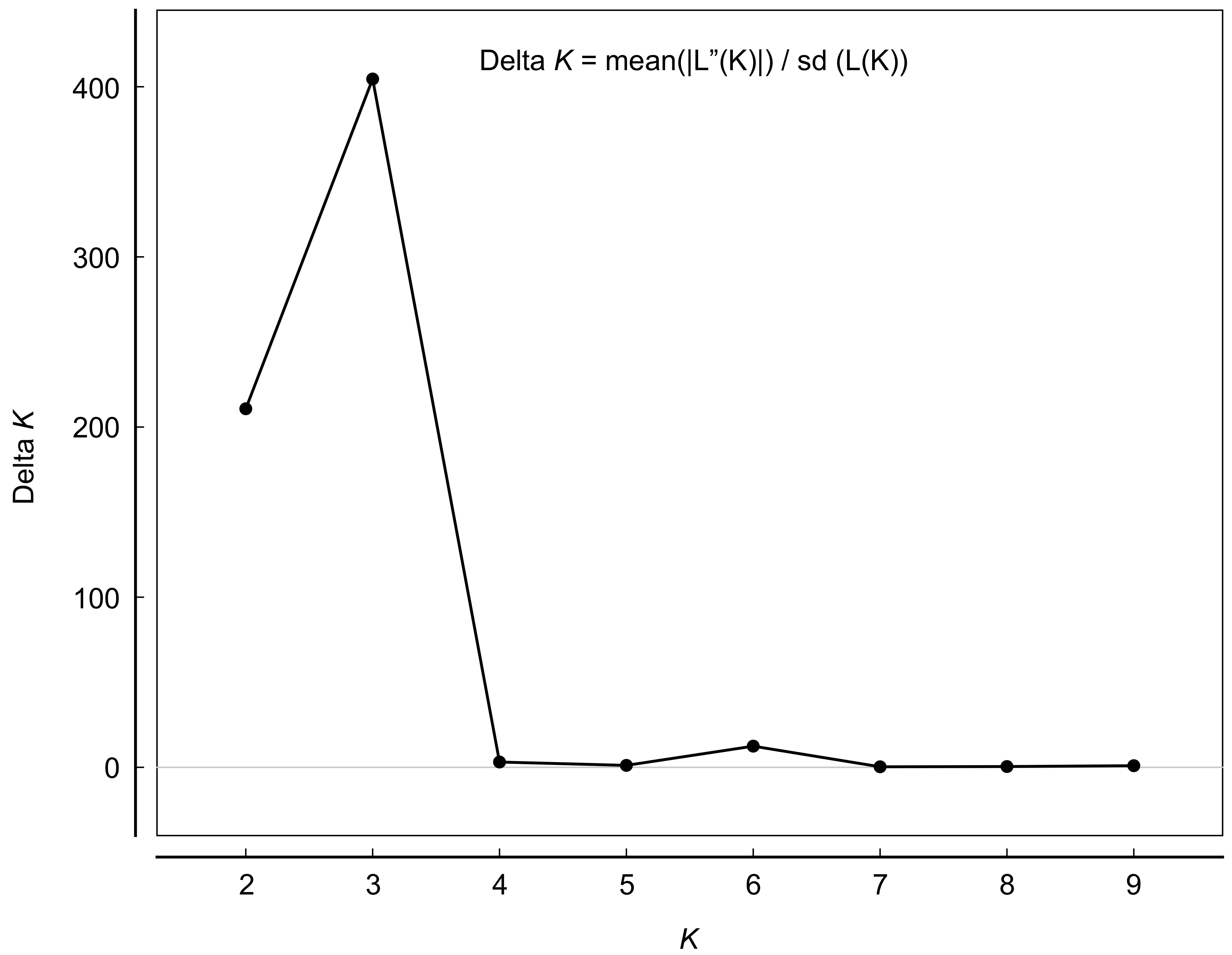

Supplement: S1 Fig — (TIF) [file pone.0203423.s001.tif]

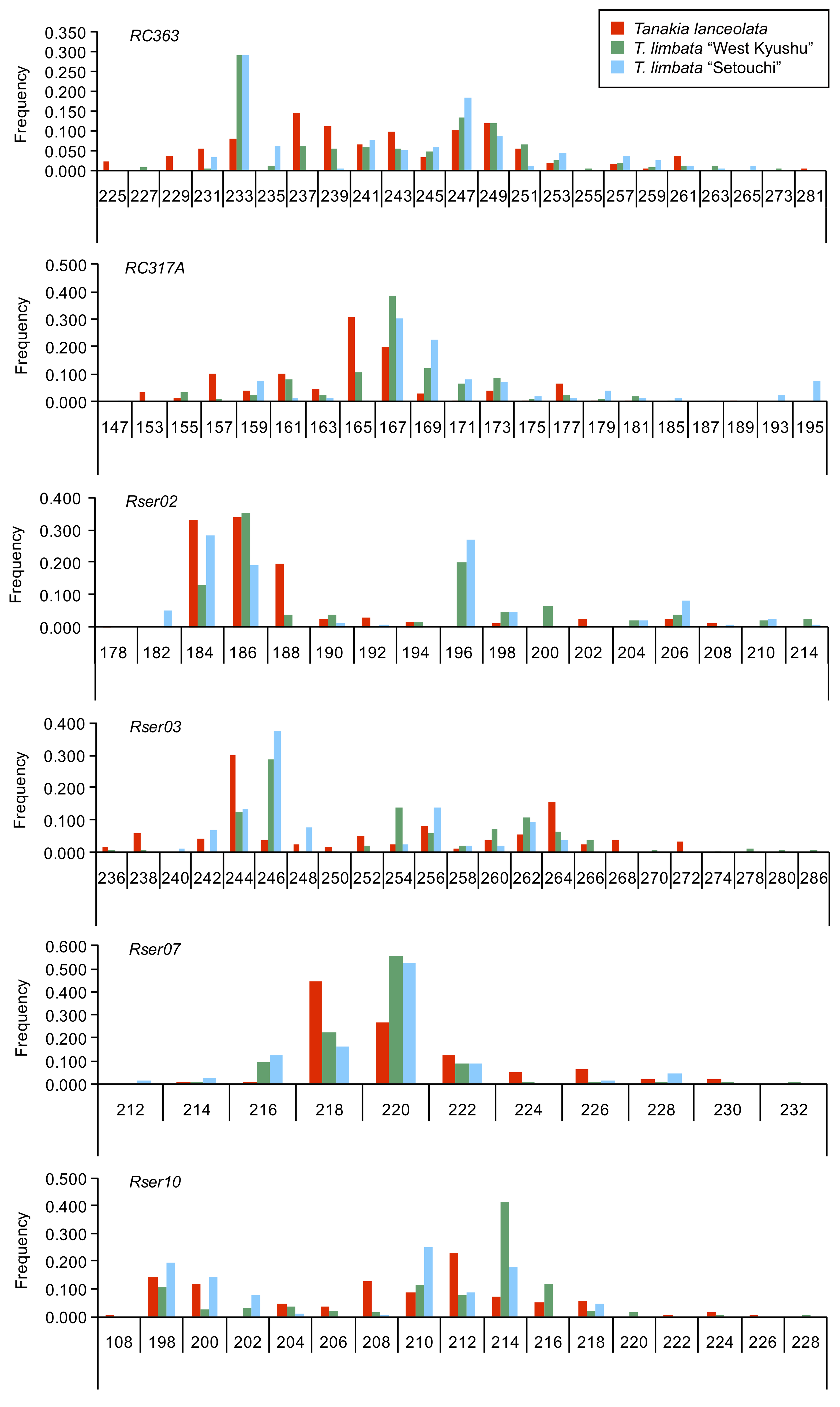

Supplement: S2 Fig — (TIF) [file pone.0203423.s002.tif]
